# *Paraburkholderia phytofirmans* PsJN-Plants Interaction: From Perception to the Induced Mechanisms

**DOI:** 10.3389/fmicb.2018.02093

**Published:** 2018-08-30

**Authors:** Qassim Esmaeel, Lidiane Miotto, Marine Rondeau, Valérie Leclère, Christophe Clément, Cédric Jacquard, Lisa Sanchez, Essaid A. Barka

**Affiliations:** ^1^Unité de Résistance Induite et Bioprotection des Plantes EA 4707, SFR Condorcet FR CNRS 3417, University of Reims Champagne-Ardenne, Reims, France; ^2^Univ. Lille, INRA, ISA, Univ. Artois, Univ. Littoral Côte d’Opale, EA 7394-ICV- Institut Charles Viollette, SFR Condorcet FR CNRS 3417, Lille, France

**Keywords:** *Paraburkholderia phytofirmans* PsJN, perception, PGPR, endophyte, biotic and abiotic stress

## Abstract

The use of plant-associated bacteria has received many scientific and economic attention as an effective and alternative method to reduce the chemical pesticides use in agriculture. The genus *Burkholderia* includes at least 90 species including pathogenic strains, plant pathogens, as well as plant beneficial species as those related to *Paraburkholderia*, which has been reported to be associated with plants and exerts a positive effect on plant growth and fitness. *Paraburkholderia phytofirmans* PsJN, a beneficial endophyte able to colonize a wide range of plants, is an established model for plant-associated endophytic bacteria. Indeed, in addition to its plant growth promoting ability, it can also induce plant resistance against biotic as well as abiotic stresses. Here, we summarized an inventory of knowledge on PsJN-plant interaction, from the perception to the resistance mechanisms induced in the plant by a way of the atypical colonization mode of this endophyte. We also have carried out an extensive genome analysis to identify all gene clusters which contribute to the adaptive mechanisms under different environments and partly explaining the high ecological competence of *P. phytofirmans* PsJN.

## Introduction

Historically, plant diseases have been controlled by the application of chemical pesticides, commonly leading to residual contamination and pathogen resistance (Couderchet, 2003). Therefore, the use of plant-associated bacteria has shown a great promise for controlling diseases and thereby reducing the use of agrochemicals in the agriculture (Pieterse et al., 2014). Plant growth-promoting bacteria (PGPB) are naturally associated with plant roots and confer positive effects to host plants (Shameer and Prasad, 2018). They can increase yield, improve plant growth, reduce pathogen infection, and enhance plants’ tolerance to adverse environmental stresses (Compant et al., 2005a). Among PGPB, endophytes are defined as those bacteria able to colonize the internal tissue of the plant without causing negative effects or external symptoms of infection on their host (Compant et al., 2008). They can enter the plant *via* different sites including tissue wounds (Agarwal, 1987), stomata (Roos and Hattingh, 1983), penetration of root hairs (Huang, 1986) and secretion of cell wall degradative enzymes (Huang, 1986; Reinhold-Hurek et al., 2006). Endophytes, some of which are belonged to the common soil bacteria, such as *Bacillus*, *Pseudomonas*, and *Burkholderia*, are applied to different plant species to promote their growth and control their diseases (Hardoim et al., 2015).

The high diverse genus *Burkholderia*, representing a group of approximately 90 species of Gram-negative β-proteobacteria (Sawana et al., 2014), has been isolated from different ecological niches including plants, soil, the atmosphere, water, fungi, animals and human (Ramette et al., 2005; Compant et al., 2008; Lackner et al., 2011; DeLeon-Rodriguez et al., 2013). Some members of *Burkholderia* have attracted a great deal of interest in biotechnology such as plant growth promotion, bioremediation, and biocontrol of plant diseases (Depoorter et al., 2016). Among *Burkholderia* strains*, Paraburkholderia phytofirmans* strain PsJN is a Gram-negative rod-shaped, non-sporulating and motile bacterium. This bacterium was first isolated from surface-sterilized onion roots infected with the mycorrhizal fungus *Glomus vesiculiferum* (Frommel et al., 1991a; Sessitsch et al., 2005). It has been recently classified as a member of *Paraburkholderia*, a group of formerly named *Burkholderia* species, mostly reported to be associated with plants and have biocontrol and bioremediation properties (Sawana et al., 2014; Eberl and Vandamme, 2016). *P. phytofirmans* PsJN has been reported as a prominent and efficient plant growth-promoting endophyte (Ait Barka et al., 2000) and a promising biological control agent against plant pathogens (Miotto-Vilanova et al., 2016).

Furthermore, *P. phytofirmans* PsJN employs different mechanisms to have a positive role in plant productivity. These mechanisms act either directly, by providing adequate plant nutrition, and producing plant hormones, or indirectly, by reducing susceptibility to diseases (Yang et al., 2009; Andreolli et al., 2016). The bacterium is also able to decrease the ethylene level in host plants through production of the 1- aminocyclopropane-1-carboxylate (ACC) deaminase enzyme (Glick et al., 2007). Moreover, it confers plants resistance against a broad spectrum of phytopathogens by the induction of plant-mediated resistance response in above ground parts of plants (Miotto-Vilanova et al., 2016). The strain also has been shown to induce tolerance toward different abiotic stresses including high temperature, cold, drought, and salinity (Bensalim et al., 1998; Ait Barka et al., 2006; Naveed et al., 2014a; Pinedo et al., 2015; Nafees et al., 2018). The complete genome sequences (8.2 Mb) of the plant endophyte *P. phytofirmans* PsJN, arranged in two chromosomes and one plasmid (121 kbp), was published by Weilharter et al. (2011). Previous comparative analysis of this strain revealed numerous biosynthetic gene clusters, secretion system, and metabolic potentials involved in endophytic behavior with diverse beneficial effects (Mitter et al., 2013; Ali et al., 2014). Compared to other PGPB, less information is available about the mechanisms attributing to biocontrol effect and endophyte lifestyle of *P. phytofirmans* PsJN, which is an established model for plant-associated endophytic bacteria. Here, together with previously published data, we summarize an inventory of knowledge on *P. phytofirmans* PsJN-plant interaction, from the perception to the resistance mechanisms associated with beneficial effects in plants. Gene clusters contribution to the adaptive mechanisms and beneficial effects on plant growth and biocontrol under different environments are also highlighted.

## Perception of *P. phytofirmans* PsJN

The interaction between endophytes and plants takes place in different areas including root and foliar surfaces as well as intercellular spaces of both root and foliar surfaces which are the first contact area for plant-associated microbes (Lindow and Brandl, 2003; Hardoim et al., 2015). A successful colonization of plant tissue by beneficial bacteria is influenced by the excretion of organic acids by their host plant (Kost et al., 2014). It has been reported that plant roots secrete a significant mixture of organic compounds known as exudates, which attract complex microbial populations present in the rhizosphere and initiate the first communication between host plants and endophytes (Kawasaki et al., 2016). The contribution of carbon sources in the recruitment of endophytic strain PsJN by host plants has been reported (Kost et al., 2014). Comparing to wild-type strain of PsJN, PsJN*Δoxc* defective in oxalate assimilation was significantly impaired in colonization of both lupin and maize and the mutant population was also significantly reduced (Kost et al., 2014). Microbial quorum sensing molecules presented by acylated homoserine lactones (AHLs) have also been shown to act as targets for host recognition and are likely implicated in communication with host plants, and subsequently colonization process (Mathesius et al., 2003). It has been shown that N-AHLs from strain PsJN play a crucial role in the communication and colonization with plants as quorum sensing mutants of PsJN could no longer efficiently colonize and promote the growth of *Arabidopsis thaliana* as compared to the wild-type (Zúñiga et al., 2013). Moreover, endophytic bacteria move toward the plant roots, using chemotactic affinity for root exudates, and subsequently followed by adhesion to the plant root surfaces. The attachment of endophytes to the root surfaces can be enhanced through the production of cellulose or exopolysaccahrides (EPS) by bacteria (Kandel et al., 2017). Furthermore, genome mining of endophytic strain PsJN revealed that many gene clusters involved in motility, biofilm production, adhesion and genes encoding for chemotactic activity and siderophore synthesis may reflect the efficient plant colonization and endophytic lifestyle of this bacterium (Mitter et al., 2013; Ali et al., 2014).

In response to microbial perception, plants evolve different strategies to recognize and respond to microbial signal exposure (Pieterse et al., 2014). The recognition is firstly achieved *via* pattern recognition receptors (PRRs) known as pathogen- or microbe-associated molecular patterns (PAMPs or MAMPs) (Jones and Dangl, 2006). To defend themselves from potential invaders, plants use PAMP-triggered immunity (PTI) as the first line of the plant immune system. Therefore, endophytes have developed strategies to minimize the plant immunity by secretion of proteins called effectors that cross the first layer of defense and suppress the PTI signaling or avoid the recognition by the host. In addition, plants have a second layer of perception in which resistance receptors mediate the recognition of effectors compounds leading to effectors-triggered immunity (ETI) which plays an essential role to control the pathogen progress (Pieterse et al., 2014).

**FIGURE 1:**
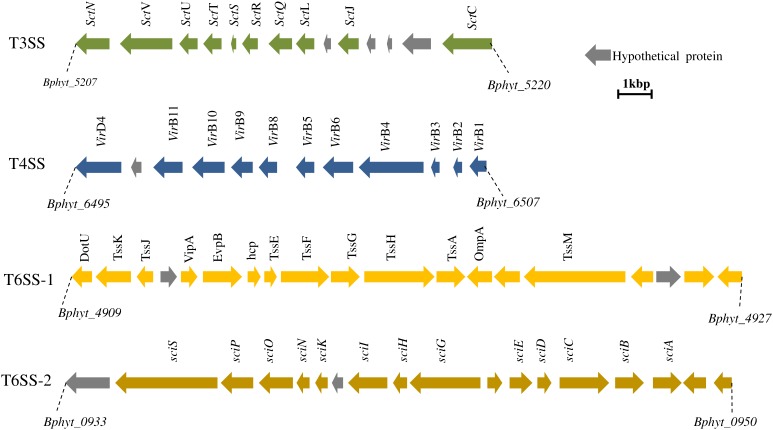
Distribution of secretion systems in the genome of *Paraburkholderia phytofirmans* PsJN. T3SS and T4SS clusters are located in chromosome 2 (position 1396242–1383596 and 2785143–2797095, respectively). T6SS is represented by two clusters located on chromosomes 1 and 2 (position 1046459–1070567 and 1046459–1058642, respectively). Secretion systems in PsJN might inject effector proteins which cross the cell wall and might act on host plant to modulate plant signal transduction and elicit host defense responses.

Plant perception of endophytic strain PsJN begins after an exposure to bacterial signals such as flagellin leading to the plant response characterized by early and long-term responses in plant immunity and plant growth regulation and morphogenesis (Bordiec et al., 2011; Trdá et al., 2013). It has been shown that *P. phytofirmans* PsJN is perceived by grapevine cell suspensions and led to the production of a monophasic and transient burst of alkalinization during the first minute of the interaction (Bordiec et al., 2011). However, no significant accumulation of H_2_O_2_ neither cell death was observed in grapevine after *P. phytofirmans* PsJN challenge (Bordiec et al., 2011; Miotto-Vilanova et al., 2016). Plant phytohormones play a crucial role as key regulators in plant defense-signaling pathways (Pieterse et al., 2012). The expression levels of related defense genes after endophyte perception is a key point as endophytes are firstly known as potential invaders. Therefore, active interference with the plant immune responses is essential for the initiation of a compatible relationship with the host plant. In case of strain PsJN, the low level of induced defense genes in the grapevine may give insight into the ability of this strain to colonize the rhizoplane and to transfer into the entire tissue leading to the endophytic behavior of this bacterium (Bordiec et al., 2011). Moreover, the distribution of different secretion system gene clusters (**Figure 1**) in the PsJN genome might play a crucial role in the plant-*P. phytofirmans* PsJN interaction (Mitter et al., 2013).

## *P. phytofirmans* PsJN Colonization and Distribution Within Plant

Endophytes are defined as those bacteria that live in plant tissues and do not visibly harm the plant (Hardoim et al., 2015). Plant colonization by endophytes can occur either in intracellular or intercellular spaces in plant tissues. The colonization depends on the strain and different colonization routes and specific interactions have been described (James et al., 2002). Some endophytic bacteria are able to penetrate the endodermis, which represents an obstacle for colonization, and inhabits the internal plant compartment (Compant et al., 2010). One of the most frequently raised questions related to endophytic bacteria is how do they enter plant tissue?

In the early stages of colonization, *P. phytofirmans* PsJN moves toward the plant roots *via* chemotactic response to plant-released compounds (Kost et al., 2014). The secretion of root exudates by the plant initiates the first communication between endophytes and host plants (Kandel et al., 2017). Moreover, quorum sensing compounds produced by strain PsJN play an important role in host communication, and the subsequent colonization process (Zúñiga et al., 2017). The attachment of endophytes to the root surfaces is considered the first colonization step which is essential in getting access to the main entry points. The production of EPS by endophytes as well as the presence of bacterial flagella and cell surface polysaccharides may help the adhesion of endophytes onto the host roots and may facilitate the colonization process (Kandel et al., 2017). Depending on the strain, endophytes can get into plant tissues through wounds, stomata, and hydathodes, which are considered as the main entry sites (Hardoim et al., 2015). The emergence zone of secondary roots and injuries constitute also a natural opening allowing the entry of the endophytes inside the plant (Kandel et al., 2017). Moreover, some bacterial endophytes deploy a wide range of catabolic activities that allow them to break down different selections of organic compounds and modify the plant cell wall compositions (Preston, 2004; Taghavi et al., 2010).

**FIGURE 2:**
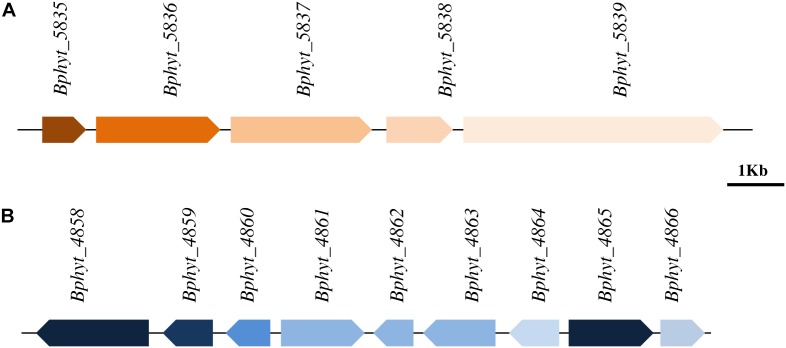
Biosynthetic genes clusters located in chromosome 2 of *P. Phytofirmans* PsJN genome. **(A)** Cellulose biosynthesis gene cluster containing the gene encoding for degradation enzyme endoglucanase *Bphyt_5838* (position 2045332–2046513) which facilitates the penetration of cell wall of host plants **(B)** genes involved in pectin degradation including genes *Bphyt_ 4858* (position 978791–980806) encoding for polygalcturonate and *Bphyt_4865* (position 988328–989917) encoding for galactarate dehydratase.

The PsJN genome harbors a total of 41 putative plant polymer genes, encoding for putative hydrolytic enzymes (Mitter et al., 2013), which may help entry into the host plant through the distraction of the host. Among them, 14 genes represent glycoside hydrolyses (GH) that are involved in cell wall and sugar metabolism. *In silico* analysis of the PsJN genome also revealed the presence of genes involved in malonate metabolizing such as malonate decarboxylase which is important for symbiosis between endophytes and the plant (Kim, 2002). Genes related to cupin superfamily involved in the modification of plant cell wall carbohydrates (Dunwell et al., 2004) are also present in the PsJN genome. Furthermore, it has been reported that bacteria, mostly related to plant pathogens, produce different extracellular enzymes or cell wall degrading factors such as cellulases, hemicellulases, and endoglucanases which enable endophytes to penetrate plant cell wall and colonize the interspatial region between plant cells (Taghavi et al., 2010; Ali et al., 2014). In the course of a search of the PsJN genome, one gene cluster (**Figure 2A**) responsible for bacterial cellulose biosynthesis has been found in the chromosome 2 and gene encoding for endo 1, 4 glucanase was found as a part of the cluster. Beside the cluster for cellulose synthesis, another one involved in pectin degradation was also found (**Figure 2B**). The latter includes genes coding for polygalcturonate and galactarate dehydratase, which are involved in the degradation of pectin and could play a crucial role in colonizing the interspatial part between plant cells (Taghavi et al., 2010). In addition, the PsJN genome harbors ABC transporter genes involved in degradation of the cell wall.

*Paraburkholderia phytofirmans* PsJN establishes rhizosphere and endophytic colonization in different plants such as potato (Bensalim et al., 1998), switchgrass (Kim et al., 2012), tomato (Pillay and Nowak, 1997), *Arabidopsis* (Zúñiga et al., 2013), maize and lupin (Kost et al., 2014), and grapevine (Ait Barka et al., 2002). The bacterium colonizes the grapevine rhizoplane immediately after the inoculation and transmits to the root interior 3 h after inoculation and then systemically migrates from the rhizoplane to aerial tissues (Compant et al., 2005b). It has been shown that the highest level of bacterial populations on grapevine rhizoplane, and aerial parts achieved at 24 and 84 h after inoculation, respectively, and the colonization level in leaves was significantly greater than stem (Compant et al., 2005b). Compant et al. (2007) showed that strain PsJN colonizes the internal xylem vessels and the bacterium takes the advantage of holes between xylem to migrate and spread to grape inflorescence stakes, pedicels, and then to youngberries. Furthermore, Mitter et al. (2017) demonstrated that *P. phytofirmans* PsJN is able to colonize the seeds of monocot and dicot after the flowers were inoculated, and subsequently get transferred into the next generation of plants. In potato plantlets, strain PsJN was observed in the first epidermal layers of roots and in the xylem tissue of stem (Frommel et al., 1991a). In maize, under normal conditions, strain PsJN was observed in root and shoot interior and the maximum population density (CFU g^-1^ dry weight) reached 5.86 × 10^5^ (rhizosphere), 5.44 × 10^5^ (root interior), and 9.36 × 10^4^ (shoot interior) (Naveed et al., 2014b). The ability of PsJN to produce EPS and form a biofilm as well as the presence of genes implicated in the biosynthesis of flagella, cellulose and genes encoding for chemotactic activity in the genome of PsJN may reflect the efficient endophytic colonization of the plant by this bacterium (Mitter et al., 2013; Ali et al., 2014). Moreover, the capacity of strain PsJN to colonize different plant hosts is probably due to large genome size (8.2 Mb), divided in two chromosomes and one plasmid (Weilharter et al., 2011).

## *P. phytofirmans* Strain PsJN, a Plant Growth Promoting Rhizobacteria

Endophytes produce beneficial effects on the plant growth through several mechanisms. The mechanism can be either direct through the production/modulation of plant hormones (Pieterse et al., 2012), facilitating resource acquisition (Naveed et al., 2014a), or indirect by producing secondary metabolites (Esmaeel et al., 2017), and induction of systemic resistance (ISR) leading to more adaptability to different stress conditions (Pieterse et al., 2014). The endophytic *P. phytofirmans* strain PsJN is a highly efficient plant beneficial bacterium as it is able to promote the growth across a range of plant species including wheat (Naveed et al., 2014a), maize (Naveed et al., 2015), brassica (Nafees et al., 2018), grapevine (Compant et al., 2005b), switchgrass (Kim et al., 2012; Wang et al., 2015), *Arabidopsis* (Poupin et al., 2013; Zhao et al., 2016), tomato (Pillay and Nowak, 1997), lupin (Kost et al., 2014), watermelon and cantaloupe (Liu et al., 1995), potato (Frommel et al., 1991a), cucumber and sweet pepper (Nowak et al., 2004). The different beneficial effects of strain PsJN on different host plants are reported in **Table 1**. The mechanisms behind the observed positive effect are linked to the production of plant phytohormones, ACC deaminase, siderophores and other secondary metabolites which contribute as signaling molecules for better bacteria-plant communication leading to an efficient colonization of plant roots (Sun et al., 2009; Naveed et al., 2015).

**Table 1:** Beneficial effects provided by the endophytic strain *Paraburkholderia phytofirmans* PsJN on different plants.

Crop	Benefit provided to the host plant	Reference
Grapevine	Growth enhancement, more secondary roots and leaf hairs.	Ait Barka et al., 2000; Compant et al., 2005b
	Increased shoot and root fresh and dry weight as well as the number of nodes.	
Maize	Increased shoot/root biomass, and leaf area.	Kost et al., 2014; Naveed et al., 2014b
	Increased leaf chlorophyll content, photosynthesis, and photochemical efficiency of PSII.	
Wheat	Better grain yield.	Naveed et al., 2014a
	Improvement of the ionic balance, antioxidant levels.	
	Increased nitrogen, phosphorus, potassium and protein concentration.	
Lupin	Degradation of plant-secreted oxalate and reduce the oxalate level which might reduce the infection potential of oxalate-producing phytopathogenic fungi or bacteria.	Kost et al., 2014
Watermelon	Root growth promotion and enhanced stem performance.	Liu et al., 1995
Switchgrass	Growth promotion.	Kim et al., 2012; Wang et al., 2015
	Increased shoot/root biomass, elongation of root, stem and leaf.	
	Early tillers and persistent growth vigor.	
	Improved the photosynthetic rates and greater water use efficiency.	
Tomato	Increased plant height, shoot/root biomass.	Frommel et al., 1991b; Pillay and Nowak, 1997; Sharma and Nowak, 1998; Nowak et al., 2004; Onofre-Lemus et al., 2009
	Greener leaves, shorter root system with more lateral roots and root hairs.	
Potato	Increased root number/dry weight, halum dry weight, stem length and node numbers.	Frommel et al., 1991a, 1993; Lazarovits and Nowak, 1997; Bensalim et al., 1998
	Induction of root branching and hair formation.	
	Increased chlorophyll and starch content, nutrient and water uptake.	
	Enhanced leaf hair formation, secondary root branching, and total plant lignin content Improved tuber number and weight, increased medium pH.	
	Enhanced tuber number and weight, earlier stolon formation.	
*Arabidopsis*	Stimulation of growth parameters (plant fresh weight, dry weight, number of root hairs and chlorophyll content)	Poupin et al., 2013; Zhao et al., 2016
	Enlarged stem cell size of pith and improved the essential metals, specifically iron, uptake and accumulation. Modulation of phytohormones.	
Cucumber	Inoculated seeds enhanced the early growth and promoted root growth and weight.	Nowak et al., 2004
Sweet pepper	Bacterized seedlings had higher initial vigor, higher root, and shoot fresh weight.	Nowak et al., 2004
Brassica	Optimized plant performance (height, root length, fresh and dry shoot biomass and root).	Nafees et al., 2018
	Improved the plant physiology parameters [photosynthetic rate, transpiration rate, stomatal conductance, chlorophyll contents (Chl), sub-stomatal CO_2_ concentration (Ci), and water use efficiency] and antioxidant activity and reduced Cr uptake in Cr-contaminated soil.	
Cantaloupe	Reduced shoot growth and root length.	Liu et al., 1995
Canola	Root elongation.	Sun et al., 2009


### 1-Aminocyclopropane-1-Carboxylate Deaminase (ACC)

The ACC, produced by endophytic bacteria, is linked with an alleviation of plant stress as it contributes to lowering the ethylene level hence promoting the plant growth (Glick et al., 1998). Indeed, ACC prevents ethylene signaling by cleaving the ethylene precursor (ACC deaminase) to ammonia and 2-oxobutanoate. Moreover, the ACC deaminase-expressing bacteria were reported to enhance the plant growth under different biotic and abiotic stresses, including pathogen attack, drought, salinity, organic and inorganic contaminants (Glick, 2004). The plant growth promoting effect in tomato associated with the strain PsJN was suggested to be linked to the expression of ACC deaminase, which plays a crucial role to enhance the growth performance of tomato plants (Onofre-Lemus et al., 2009). Sun et al. (2009) showed that ACC produced by strain PsJN is involved in the colonization as the deletion mutant *P. phytofirmans* PsJN Δ*acd*S (*Bphyt_5397*) lost its ability to promote the elongation of the roots of canola seedlings.

### Production of the Plant Growth Promoting Hormones Indole-3-Acetic Acid (IAA)

The production of IAA by endophytic bacteria has received a lot of attention due to its crucial role in each stage of the plant development (Pieterse et al., 2012). Complete genome analysis of strain PsJN revealed the presence of relevant genes involved in the indole-3-acetamide and the tryptophan side chain oxidase pathways (Weilharter et al., 2011). The production of IAA by PsJN was experimentally demonstrated with or without the addition of L-ryptophan (L-TRP), a precursor of auxins in plants (Naveed et al., 2015). Furthermore, this study demonstrated that the different plant growth parameters (plant height and biomass, photosynthesis, and chlorophyll content) of maize were significantly improved when applying PsJN inoculum supplemented with L-TRP. It has been previously shown that production of IAA by strain PsJN is likely involved in the efficient colonization of *Arabidopsis* (Zúñiga et al., 2013). The ability of strain PsJN to produce and degrade IAA as a sole carbon source gave an insight into the ability of this bacterium to resist under different stresses and lead to understand its friendly interaction with host plant (Donoso et al., 2017). Beside its role in the colonization, the IAA is also involved in the root proliferation (Zúñiga et al., 2013) and is considered as a signaling molecule in bacteria-plant interactions (Van Puyvelde et al., 2011).

### Siderophore Production and Other Secondary Metabolites

**FIGURE 3:**
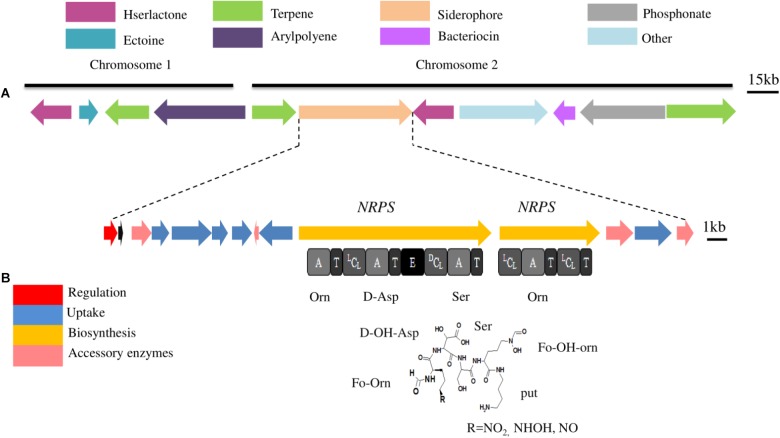
**(A)** Secondary metabolite gene clusters predicted in the genome of *P. phytofirmans* PsJN. These gene clusters have been predicted using Florine workflow (Esmaeel et al., 2016b). Each color of gene clusters represents a different class of metabolites. Clusters were located in chromosomes 1 or 2 and some clusters are present in both chromosomes. **(B)** Siderophore gene cluster located in chromosomes 2. Gene implicated in the synthesis are represented by arrows. The domain organization of the NRPS genes is shown below arrows. A, Adenylation domain; ^L^C_L_, condensation between 2 L-monomers; ^D^C_L_, condensation between D-monomer and L-monomer; E, Epimerization domain; T, Thiolation domain. Predicted amino acid specificity is shown under each A domain, Asp, aspartic acid; Ser, serine; hfOrn, hydroxyformylornithine.

Iron is essential for microorganisms due to its intervention in the synthesis of many essential components of the cell (Leclère et al., 2009). The availability of iron in the environment to living microbes is very low due to its poor solubility at neutral pH 7 (Andrews et al., 2003). Therefore, bacteria including endophytes have evolved several pathways including siderophore with high affinity to scavenge and transport iron from the environments (Loaces et al., 2011; Esmaeel et al., 2016a). Beside their ability to produce siderophores, some endophytes have membrane receptor proteins for the uptake of siderophores produced by other endophytes (Cornelis and Bodilis, 2009). The ability of endophytes to produce or capture siderophores, under iron stress condition, is one the most traits which provide iron to host plants (Johnson et al., 2013). It also contributes to protect host plant against phytopathogenic infection (Miethke and Marahiel, 2007), to activate the ISR (Van Loon et al., 2008), to facilitate the bacteria-plant interaction and is involved in the colonization of root, stem, and leaves (Compant et al., 2005a). The main mechanism of siderophore synthesis is achieved through modular megaenzymes called non-ribosomal peptides synthetases (NRPSs), and others are assembled by various enzymes known as NRPS independent siderophore (NIS) (Challis, 2005). NRPSs organized in modules, arranged in sets of primary domains including adenylation (A), thiolation (T), condensation (C), and thioesterase (TE) domains, which synthesize basic peptides. Moreover, secondary domains such as epimerization (E) are also contributing in the modifying peptides into more structurally complex peptides (Esmaeel et al., 2016b). The genome of *P. phytofirmans* PsJN was mined with the aim to screen all potentially produced secondary metabolites (SMs), especially those produced by NRPSs by following Florine workflow previously described (Esmaeel et al., 2016b) (**Figure 3A**). Genome analysis of PsJN revealed the presence of a siderophore gene cluster located in the chromosome 2 (*mba*A to *mba*N). This cluster includes two NRPS genes (*mba*A and *mba*B) as well as genes involved in uptake and accessory genes (**Figure 3B**). The cluster also includes a gene (*mba*F) encoding for an extracytoplasmic sigma 70 factor involved in the regulation. Furthermore, the strain PsJN is also possesses 7 outer membranes ferric related siderophore receptors (TonB dependent), which are essentially required for strain PsJN to compete for iron through the uptake of siderophore-iron complexes produced by other microorganisms.

## *P. phytofirmans* Strain PsJN, a Plant Defender

The high demand for agriculture crops is growing and is expected to keep on increasing for decades (Tilman et al., 2011). Under stress conditions, including biotic and abiotic, plants can be subjected to negative effects on the growth resulting in heavy losses. Therefore, improvement of plant tolerance to stress using plant-associated bacteria has arisen as an alternative strategy to enhance plant adaptation to different stress conditions (Shameer and Prasad, 2018). While most of the described endophytes protect the plant from biotic stresses, some are also able to defend their host plants against abiotic stresses. Among them, the endophytic strain PsJN that has been shown to improve tolerance toward high temperature, heavy metals (Nafees et al., 2018), cold, drought, and salinity stresses (Ait Barka et al., 2006; Naveed et al., 2014a; Pinedo et al., 2015) (**Table 2**). The role of strain PsJN on plants stress tolerance is discussed in the following subsections.

**Table 2:** The proposed mechanism behind elevation in stress tolerance of different plants provided by the endophytic strain *P. phytofirmans* PsJN.

Stress conditions		Host	Mechanisms associated to the tolerance	Reference
**Biotic stresses**	*Botrytis cinerea*	Grapevine	Biofilm formation around *Botrytis.*	Ait Barka et al., 2000; Miotto-Vilanova et al., 2016
			Induced the expression of defense related genes (*PR1*, *PR2*, *PR5*, and *JAZ*).	
			Modulated the level of leaf carbohydrate and chlorophyll fluorescence.	
	*Verticillium dahliae*	Tomato	Reduced the severity of *Verticillium* wilt in tomato through induction of defense response.	Sharma and Nowak, 1998
			Improved the performance of plant which help plant to be more efficient to endure and reduce disease severity.	
	*Fusarium oxysporum* sp. *lycopersici*	Tomato	PsJN combined with *Serratia plymuthica* increased the resistance of tomato plants co-cultured with *Fusarium oxysporum* sp. *Lycopersici.*	Frommel et al., 1991b
	*Pseudomonas syringae*	*Arabidopsis*	Activation of plant defense-signaling pathways (salicylic acid, jasmonate, and ethylene).	Su et al., 2017; Timmermann et al., 2017
			Primed the expression of plant defense-related genes (PR1, PDF1.2).	
**Abiotic stresses**	High temperature	Potato	Induced the synthesis of tuberization factors, jasmonic acid which compensate abscissic acid (ABA).	Lazarovits and Nowak, 1997; Bensalim et al., 1998
			Induced morphological, physiological and cytological modifications represented by sturdier stem, larger leaves, more leaf hairs, more plastid numbers, more functional stomata leading to greater efficiency in controlling water loss.	
	Low temperature	Grapevine	Enhanced CO2 fixation and O2 evolution.	Ait Barka et al., 2006; Theocharis et al., 2011; Fernandez et al., 2012
			Accumulated the stress-related metabolites such as starch, proline, and phenolics and increased levels of soluble sugars (glucose, fructose, saccharose, M6P the precursor of mannose, raffinose, and maltose).	
			Enhanced the expression of antifreeze related genes (PR proteins), cold-specific transcription factor CBF4, stilbene synthase (STS), phenylalanine ammonia-lyase (PAL) and LOX genes.	
	Drought stress	Maize And wheat	Increased the leaf water content by 30%.	Naveed et al., 2014a,b
			Reduced leaf damage.	
			Improved the morphological and physiological (photosynthetic rate, water use efficiency and chlorophyll content) performance of plant.	
			Enhance water uptake.	
			Improved the ionic balance, antioxidant levels, and also increased the nitrogen, phosphorus, potassium and protein concentrations in the grains.	
	Salt stress	*Arabidopsis*	Accumulated less sodium within leaf tissues.	Pinedo et al., 2015; Ledger et al., 2016
			Accelerated the accumulation of proline, ROS scavenging, detoxification, and expression of abscisic acid signaling pathway, and down-regulated the expression of jasmonic acid biosynthesis related genes.	
			Regulated the expression of important ion-homeostasis related genes.	
			Production of ACC, auxin catabolism, *N*-acyl-homoserine-lactone production, and flagellin synthesis.	
	Heavy metal contaminated soil	Brassica	Stabilized chromium (Cr) levels in soil and reduced Cr uptake in Cr–contaminated soil.	Nafees et al., 2018


### Plants Under Biotic Stress

Most of endophytic bacteria are well known for their capacity to produce secondary metabolites that have an inhibitory effect toward a wide range of phytopathogens. These metabolites comprise polyketides, non-ribosomal peptides, terpenoids, alkaloids, steroids, flavonoids, 2-phenylethanol, and phenols (Taghavi et al., 2010; Hardoim et al., 2015; Esmaeel et al., 2017). Some of these compounds are important for protection and also play a significant role in mechanisms of signaling, defense, and genetic regulation of the interaction (Shameer and Prasad, 2018). *P. phytofirmans* PsJN has been reported as a remarkable biocontrol agent against *Botrytis cinerea* on grapevine (Ait Barka et al., 2000), *Fusarium oxysporum* and *Verticillium dahliae* on tomato (Frommel et al., 1991b; Sharma and Nowak, 1998), and *Pseudomonas syringae* on *Arabidopsis* (Su et al., 2017; Timmermann et al., 2017) (**Table 2**). Inoculation of the grapevine (*Vitis vinifera* L.) plantlets with PsJN induced their resistance against the pathogen *B. cinerea*, achieving a significant reduction of *Botrytis*-related necrosis (Ait Barka et al., 2000; Miotto-Vilanova et al., 2016). The mechanism behind the protection of *in vitro-* PsJN-bacterized plantlets against the mold disease can be explained through the direct inhibition of spore germination and disruption of the cellular membrane hence inducing mycelium cell death (Ait Barka et al., 2002; Miotto-Vilanova et al., 2016). Previous studies of plant-microbe interactions have discussed the ability of PGPB to trigger plant immune response and induce the systemic resistance (ISR) (Pieterse et al., 2014). Several factors have been identified to be responsible for ISR including flagella, antibiotic, *N*-acyl-homoserine lactones, lipopolysaccharides, salicylic acid (SA), jasmonic acid (JA), siderophores, and volatile compounds (Van Loon et al., 2008; Bordiec et al., 2011). Miotto-Vilanova et al. (2016) showed that strain PsJN-induced resistance against *Botrytis* is clarified through the induction of plant immunity response. When the bacterium is perceived by the plant cell, no significant induction of plant-related genes was observed. However, after the pathogen challenge, PsJN primed the expression of SA and JA related genes; modulated the level of leaf sugars and accumulated the stress-related metabolites including H_2_O_2_ and callose deposition (Miotto-Vilanova et al., 2016).

In tomato, bacterized plants challenged with *V. dahliae* caused a significant increase in plant height and biomass resulting in better performance of plant to withstand and reduce the severity of *Verticillium* wilt disease (Sharma and Nowak, 1998). Moreover, the combination of strain PsJN and *Serratia plymuthica* induced the resistance of tomato plants co-cultured with *Fusarium oxysporum* sp. *lycopersici* (Frommel et al., 1991b).

In *Arabidopsis*, the strain reduced disease severity and incidence of *Pseudomonas syringae* pv. *Tomato* DC3000 (Timmermann et al., 2017). In this study, strain PsJN-treated plants exhibited resistance to pathogen infection *via* the activation of plant signaling pathways including salicylic acid, jasmonate, and ethylene, leading to higher expression of plant defense-related genes. In the other hand, Su et al. (2017) showed that PsJN, *in vitro*, did not exhibit direct antibacterial activity toward *P. syringae*. However, *in planta*, the presence of PsJN at the site of infection alleviates the pathogen growth during the early stage of infection. In addition, bacterized seeds limited the presence of the pathogen in the root system through priming the expression of plant defense-related genes.

### Plants Under Abiotic Stress

Plants are exposed to different environmental stresses such as drought, heat, heavy metals, and salinity which have profound effects on plant growth and yield leading to significant reduction in crops production. Thus, the reduction of abiotic stresses using eco-friendly strategy is essentially important. One way to achieve the sustainable agriculture and reduce the loss is the application of beneficial bacteria, which provides better choice to improve the crop productivity and enhance the plant tolerance to different stresses. The use of *P. phytofirmans* strain PsJN to alleviate and induce tolerance toward different abiotic stresses in different crops (**Table 2**) has been reported, hence opening an effective and a promising strategy for sustainable agriculture.

#### Heat Stress

The high temperature is a serious problem affecting plant physiology and maturity leading to enormous crop losses. In the field, plant growth and quality are affected by different stresses including high temperature surrounding the plants. Since each species has an optimum range of temperature, temperatures exceeding this range would initiate a heat stress which is a primary factor imposing a drastic impact on plant growth resulting therefore in heavy losses (Hatfield et al., 2011). As a result of global climate change, the rate of high temperature is expected to keep on increasing in different parts of the world which negatively influences the yield. High temperature causes a reduction in roots and shoots development, a severe reduction in potato tuber number and fresh weight (Bensalim et al., 1998). Therefore, evolving low-cost strategies to enhance the plant tolerance to heat stress would help in overcoming the negative impact of climate change. The use of PGPB to improve the tolerance to elevated temperature has received a lot of attention as a promising method for sustainable agriculture. In potato, the effect of inoculation with strain PsJN on the plant growth at elevated temperature was reported (Bensalim et al., 1998). As compared to non-PsJN treated (control), *in vitro*-plantlet inoculated with strain PsJN showed a better performance in plant growth likely due to the development of more secondary roots (Frommel et al., 1991a) which lead to more water and nutrient availability. The ability of the PsJN strain to accumulate cytokinin content and increase medium pH in bacterized plantlets might also explain its significant role to modify the plant performance and consequently increase heat stress tolerance (Lazarovits and Nowak, 1997).

#### Low Temperature

Plant health can be subjected to different environmental stresses including low temperature which affects the geographical distribution of many plant species and causes significant impacts in the yield of the most valuable agricultural crops (Theocharis et al., 2012). Under low temperature, plants evolve several physiological and molecular changes to improve their tolerance to cold stress. This process is known as cold acclimation which includes different modifications such as accumulation of carbohydrates and osmolytes, the expression of stress-related genes, and specific proteins synthesis (Ruelland et al., 2009; Theocharis et al., 2012). The use of beneficial bacteria to enhance chilling resistance has been reported as a new solution to induce plant defense to cope toward cold stress (Yang et al., 2009; Theocharis et al., 2011).

The *P. phytofirmans* strain PsJN was shown to help plants to overcome chilling stress in the grapevine by inducing physiological and biochemical changes. PsJN-bacterized plantlets showed significant elevation of proline, phenolics, starch deposition, and the photosynthetic rate was also enhanced as compared to non-PsJN treated plantlets (Ait Barka et al., 2006). Furthermore, bacterized plantlets exhibited significant accumulation of compatible osmolytes which are involved in cold adaptation in plants (Theocharis et al., 2011). Moreover, Fernandez et al. (2012) demonstrated that grapevine-bacterized plantlets, upon exposure to low temperature, accumulated more concentrations of starch, raffinose, and mannose, and other soluble sugars, likely related to the stimulation of reactive oxygen species (ROS)-scavenging system by this bacterium (Theocharis et al., 2011). The PsJN-associated tolerance to low-temperature stress might also be related to enhanced expression of stress-related genes stilbene synthase and phenylalanine ammonia-lyase, involved in the synthesis of resveratrol and SA, respectively, known for their involvement in plant stress responses (Theocharis et al., 2011, 2012). Moreover, PsJN enhanced the expression of cold-specific transcription factor CBF4 and PR proteins, including acidic chitinases and a basic glucanase, both are well known for their involvement in grapevine resistance against pathogen attacks (Miotto-Vilanova et al., 2016) and cold stress (Griffith and Yaish, 2004).

#### Drought Stress

Drought is a major abiotic stress that limits the plant productivity. It affects plant growth and crop quality by reducing plant water availability leading to physiological and morphological changes such as leaf wilting, reduction in chlorophyll content, root elongation and production of ROS (Vurukonda et al., 2016). To cope with this stress, different solutions have been reported including the use of PGPB as a promising strategy to improve plant growth under water deficit conditions (Yang et al., 2009). The inoculation of plants with strain PsJN led to growth improvement, increased nutrient uptake and helped plants to be more tolerant to drought stress (Naveed et al., 2014a). In maize (*Zea mays* L.), under drought stress conditions, inoculation with strain PsJN resulted in more production of plant biomass and significantly improved the physiological traits in both varieties comparing to plant inoculated with *Enterobacter* sp. FD17 or non-inoculated plant (control) (Naveed et al., 2014b). In wheat (*Triticum aestivum* L.), under reduced irrigation, strain PsJN was effective to improve relative water content, chlorophyll content, antioxidant activities, and photosynthetic rate, and consequently improved crop yield and quality (Naveed et al., 2014a). The mechanism of drought-stress tolerance could be explained by the alteration of plant defense-related genes in the presence of strain PsJN (Sheibani-Tezerji et al., 2015).

#### Salt Stress

Salinity is a serious threat affecting the plant productivity worldwide (Gupta and Huang, 2014). It defines by the accumulation of excessive amounts of sodium salts in the plant tissue. As a result of salinity stress, plant can be subjected to significant physiological disorder (s) leading to substantial loss in crop productivity and quality. The most drastically step of increased salinity is the ion imbalance resulting in high Na^+^ concentration and consequently leading to deleterious effects on cell metabolism. Furthermore, accumulation of Na^+^ at high concentration minimizes the plant’s ability to take up K^+^ ions, an essential macroelement for proper growth and development of plants (Gupta and Huang, 2014). It is involved in many plant processes such as protein synthesis, the activation of enzymes, cell metabolism, photosynthesis, and osmoregulation. Furthermore, for osmotic functions, most plants have preference for K^+^ rather than Na^+^ (Zhang et al., 2009). Therefore, under salinity stress, plants require sufficient amounts of K^+^ ions that are captured by the root from the soil particles. The accumulation of Na^+^ can be influenced by the presence of other elements. For example, the supplement of Ca^2+^ and Mg^2+^ was shown to have a protective effect on salinity stress through the inhibition of Na+ transport (Zhang et al., 2009).

As a result of osmotic stress, different physiological disorders are generated including accumulation of ROS, reduced photosynthetic capability, increased ethylene, and reduction of root and shoot length (Zhang and Shi, 2013; Deinlein et al., 2014). Therefore, the improvement of plant salinity tolerance is urgently needed to cope the growing demand for crop production. The use of plant growth-promoting bacteria as a valuable strategy to promote the salt stress tolerance in plants have been reported (Han et al., 2014). Plants inoculated with beneficial bacteria were shown to increase their tolerance toward salinity stress by maintaining the K^+^/Na^+^ ratio and reducing the sodium salts in the cytoplasm (Zhang and Shi, 2013; Gupta and Huang, 2014).

In *A. thaliana*, upon exposure to salt stress, plants treated with *P. phytofirmans* strain PsJN displayed different salt-stress responses involved in ROS scavenging and ABA-dependent pathways (Pinedo et al., 2015). PsJN-Inoculated plants induced the expression of genes involved in ion homeostasis and one gene associated to JA biosynthesis was down regulated. The mechanism of salt-stress tolerance associated with PsJN could be explained by the priming effects of the strain and maintaining the expression of salt stress-related genes over time. Beneficial bacteria can also induce the tolerance by the secretion of exudates osmolytes which can act together with other osmolytes produced by plants to reduce the negative effects of salt stress and stimulate plant growth (Paul and Nair, 2008). The volatile compounds (VOCs) produced by PsJN were shown to play a crucial role in the plant growth promoting effect and tolerance to salinity stress. Exposure of *A. thaliana* to VOCs produced by PsJN induced the growth promotion and the salt-stress tolerance (Ledger et al., 2016).

## Conclusion

Plants are exposed to different environmental stress conditions that reduce their productivity. While no real solutions are available to withstand abiotic stress, chemical pesticides are mainly applied to control plant pathogens and to enhanced crop yield and quality. However, negative impacts such as a development of resistance in pathogenic races are generated. Therefore, one strategy to minimize the use of synthetic fungicides is the application of beneficial microbes able to improve the plant health and enhance the plant defense against a broad range of phytopathogens.

**FIGURE 4:**
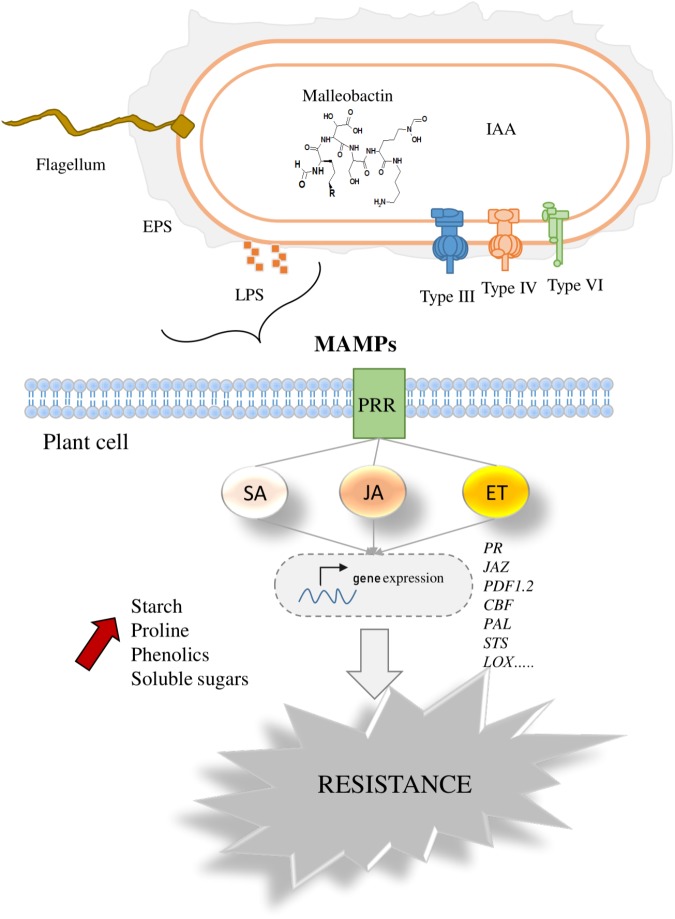
Overview of plant perception of *P. phytofirmans* PsJN. The figure explains the range of signals produced by strain PsJN that can be perceived by host plant cell. These include flagellin, phytohormones, malleobactin, LPS, and effectors proteins, injected by secretion pathways. After exposure to bacterial signals, plant cell responses are characterized by early and long-term responses in plant immunity represented by the expression of plant defense-signaling networks and modulation the level of soluble sugars, starch, proline, and phenolic compounds. PR, pathogenesis-related proteins; JAZ, jasmonate; PDF1.2, plant defensin; PAL, phenylalanine ammonia lyase; STS, stilbene synthase; LOX, lipoxygenase; EPS, Exopolysaccahrides; IAA, indole-3-acetic acid; Type III, type III secretion system; Type IV, type IV secretion system; Type IV, type VI secretion system.

In this review, the potential beneficial effects of *P. phytofirmans* strain PsJN on different host plants have been highlighted. The bacterization with the strain PsJN improved the growth parameters in different host plants and significantly reduced the negative impacts of biotic and abiotic stresses. Studies of plant-*P. phytofirmans* PsJN interaction have discussed different key signals implicated in plant perception of the bacterium and in the bacterial modulation of host metabolisms, which help to understand the observed positive effect of strain PsJN on host plants (**Figure 4**). Furthermore, the positive roles of strain PsJN in sustainable agriculture emphasized its promising applications to optimize crops performance and improved their tolerance to different environmental stress conditions.

Overall, beneficial effects associated with strain PsJN suggest using this bacterium as a model system in sustainable crop production. This will open new doors for improving plant health and reducing the global dependency on chemical pesticides.

On the other side, beneficial effects of plant-associated bacteria vary under artificial laboratory conditions, greenhouse, and field trials. The intended results under field trails are sometimes difficult due to the unpredicted environments as well as the climate variation which impact the effectiveness of plant associated bacteria. Furthermore, in the field, the survival and the viability of bacterial cell need to be more explored.

## Author Contributions

QE did the writing. QE and LS drew the graphs. LM and MR were partially involved in writing the review. VL, CC, CJ, LS, and EB revised the manuscript with contributions and discussion from all co-authors. All authors given their approval to the final version of the manuscript.

## Conflict of Interest Statement

The authors declare that the research was conducted in the absence of any commercial or financial relationships that could be construed as a potential conflict of interest.
